# Putting health metrics into practice: using the disability-adjusted life year for strategic decision making

**DOI:** 10.1186/1471-2458-13-S2-S2

**Published:** 2013-06-17

**Authors:** Kim Longfield, Brian Smith, Rob Gray, Lek Ngamkitpaiboon, Nadja Vielot

**Affiliations:** 1 Population Services International, 1120 19th Street NW, Suite 600, Washington, DC, 20036; 2 Population Services International/Laos, T4 Road, Unit 16, Donkoi Village, Vientiane, Lao PDR, PO Box 872

## Abstract

**Background:**

Implementing organizations are pressured to be accountable for performance. Many health impact metrics present limitations for priority setting; they do not permit comparisons across different interventions or health areas. In response, Population Services International (PSI) adopted the disability-adjusted life year (DALY) averted as its bottom-line performance metric. While international standards exist for calculating DALYs to determine burden of disease (BOD), PSI's use of DALYs averted is novel. It uses DALYs averted to assess and compare the health impact of its country programs, and to understand the effectiveness of a portfolio of interventions. This paper describes how the adoption of DALYs averted influenced organizational strategy and presents the advantages and constraints of using the metric.

**Methods:**

Health impact data from 2001-2011 were analyzed by program area and geographic region to measure PSI's performance against its goal of doubling health impact between 2007-2011. Analyzing 10 years of data permitted comparison with previous years' performance. A case study of PSI's Asia and Eastern European (A/EE) region, and PSI/Laos, is presented to illustrate how the adoption of DALYs averted affected strategic decision making.

**Results:**

Between 2007-2011, PSI's programs doubled the total number of DALYs averted from 2002-2006. Most DALYs averted were within malaria, followed by HIV/AIDS and family planning (FP). The performance of PSI's A/EE region relative to other regions declined with the switch to DALYs averted. As a result, the region made a strategic shift to align its work with countries' BOD. In PSI/Laos, this redirection led to better-targeted programs and an approximate 50% gain in DALYs averted from 2009-2011.

**Conclusions:**

PSI's adoption of DALYs averted shifted the organization's strategic direction away from product sales and toward BOD. Now, many strategic decisions are based on "BOD-relevance," the share of the BOD that interventions can potentially address. This switch resulted in more targeted strategies and greater program diversification. Challenges remain in convincing donors to support interventions in disease areas that are relevant to a country's BOD, and in developing modeling methodologies. The global health community will benefit from the use of standard health impact metrics to improve strategic decision making and more effectively respond to the changing global burden of disease.

## Background

Program impact and the cost effectiveness of investments in health interventions are pressing concerns among global health organizations, due to an environment of decreasing resources. Donors and implementing organizations are increasingly asked to demonstrate value for money, prompting the use of health metrics to show quantifiable results. Donors use health metrics to monitor and evaluate the performance of their recipients and to inform their investments. Implementing organizations use metrics to measure program success, document best practices, and report back to donors on performance. The larger global health community - which includes donors and implementers as well as researchers and policy makers - uses metrics to inform its discussions and identify each stakeholder's role in health promotion.

The pressure on implementing organizations to be accountable for performance is increasing. Recently, the United States Agency for International Development, the Global Fund, the United Kingdom's Department for International Development, and the World Bank have adopted performance-based funding systems to reward programs that deliver positive health outcomes, while reducing aid to organizations that do not [1-4]. To prioritize program development and improve decision making, standard measurement processes and agreement on which health metrics best address the majority of the global health community's concerns are needed [5-7].

In the past, health impact has typically been expressed through metrics such as cases averted and deaths averted for interventions preventing disease acquisition or progression. Calculations for cases and deaths averted are disease specific and can be applied to any health area where the outcome includes mortality, including HIV/AIDS, sexually transmitted infections (STIs), diarrheal diseases, maternal health, and malaria [8-10]. Another metric, couple-years of protection (CYPs), has been used to estimate the impact of FP products and services [11,12]. While useful for disease-specific inquiries, these metrics pose a major challenge for priority setting: they do not permit comparisons across different interventions or health areas to inform decision making based on potential health impact.

Summary measures of population health (SMPH) are more comprehensive than the aforementioned metrics: they provide convenient, single-unit snapshots of a particular health situation in a given context. Murray and Lopez identified eight uses of SMPH for improved decision making: comparing health status across various populations; comparing health status of the same population over time; identifying health inequalities among populations; accounting for the burden of nonfatal health outcomes and not simply mortality; setting priorities for health services improvements; setting priorities for health research and development; improving public health training curricula; and analyzing the cost-effectiveness of health promotion programs [13,14]. SMPH combine all relevant health information about a population into a bottom-line measure that can be used with stakeholders [15].

This paper focuses on one particular SMPH, the disability-adjusted life year. The World Bank and World Health Organization (WHO) developed the DALY in 1993 to address the need for SMPH that account for mortality and morbidity, as well as provide objective information about population health status for decision making [7,16,17]. DALYs have been preferred to other "gap measures" because they allow morbidity and mortality to be disaggregated into years of life lost and years of life lost due to disability [18]. The result is a metric that combines the value of lives saved with illness and disability prevented.

The most common use of DALYs is to estimate the BOD in a population. The WHO Global Burden of Disease uses DALYs to demonstrate where certain diseases are concentrated and where health areas have been disproportionately addressed, particularly at the national level [19,20]. DALYs are a convenient unit of measurement because researchers and decision makers can disaggregate results by region, sex, age group, and disease type. Estimation of health burden using DALYs also enables users to detect key differences across populations [21,22]. Researchers and implementers frequently apply DALYs to estimate BOD for health conditions perceived as neglected in order to garner funding and program support from donors and policy makers [23-29].

Like other metrics, DALYs can also be used along with cost data to estimate the cost-effectiveness of health interventions by simply dividing overall costs by health impact, the number of DALYs averted by the intervention. Cost-effectiveness is a critical input to strategic decision making, particularly when interventions are life saving, but costs vary greatly between strategies. Cost-effectiveness analyses using cost per DALY averted have become standard for health programs in low- and middle-income countries [30-42].

This paper describes why one international non-governmental organization (INGO), Population Services International, adopted the DALY as its bottom-line estimate for health impact. It describes how the organization uses DALYs averted for strategic decision making at the global, regional, and country levels, and relates how the adoption of this metric had a profound impact on PSI's strategic direction. The paper also presents the advantages and practical constraints of using DALYs averted to optimize an organization's health impact and offers considerations for using the metric in the future.

### PSI's history of health impact measurement

PSI is an INGO that uses social marketing to improve the health status of the poor and vulnerable in developing countries. By adapting marketing strategies and methods developed in the for-profit sector, PSI helps those in need adopt healthy behaviors. PSI has also modeled its management practices on the for-profit sector, creating an organizational culture where there is a strong focus on its bottom line, a discrete set of metrics that quantify organizational achievement. Since performance management goals for programs, as well as individual staff, are tied to this organization-wide measure, program staff actively work to maximize their contributions and increase the bottom line.

For its first two decades, PSI measured its bottom line by the number of products sold. Since the organization worked primarily on FP, sales of contraceptives were also converted into CYPs. During these early years, many meetings among senior decision makers revolved around the monthly sales report, which compiled activities from all PSI interventions. While reviewing sales reports is a simplistic way to view programmatic progress by today's standards, the process did help identify areas of high and low performance. More importantly, this use of metrics helped the organization think more strategically by looking beyond individual intervention deliverables to focus on building "programs," portfolios of interventions that would deliver value over the long term.

In the 1980s and mid-1990s, PSI introduced other programs into its portfolio, including diarrheal disease (early 1980s), HIV/AIDS (late 1980s), and malaria (early 1990s). In the late 1990s, PSI adopted Person-Years of Protection (PYP), a new, simple conversion factor, to estimate the number of person-years protected by the number of health product units sold or distributed. While the PYP allowed PSI to create an aggregate bottom-line measure, its utility was limited. The PYP did not estimate health impact; rather, it expressed sales using a common denominator for interventions of different durations. Therefore, this metric did not allow decision makers to see differences in the potential health impact across interventions. It also did not include estimates of effectiveness and gave equal weight to all products distributed. Finally, the PYP was specific to PSI, which did not allow the organization to compare its performance to that of other implementation organizations.

To address the limitations of PYPs, PSI adopted DALYs averted as its key performance metric in 2006. DALYs averted counts the number of DALYs that are not lost, but averted, by a health intervention. By using DALYs averted, the organization could estimate the health impact of its products, services, and behavior change interventions across all of its health areas. These results, in turn, would inform strategic decision making. While international standards existed to calculate DALYs at the national level for determining BOD, PSI's use of DALYs averted was novel. It used DALYs averted to measure the effectiveness of a portfolio of interventions and to estimate the health impact of those interventions.

Basing a metric on the DALY offered several advantages for estimating organizational health impact. The DALY is a recognized unit for measuring BOD, which ensures alignment with the international public health community. It also enables PSI to measure the impact of all of its interventions and in relation to BOD, which rewards and reinforces targeted interventions. PSI can combine this impact with cost for internal comparisons of its interventions' cost-effectiveness as well as to compare its costs with other INGOs and against global standards. Additionally, the DALY permits comparisons of impact across different health conditions and across countries.

It is important to note that while the DALY captures information on maternal and child mortality and morbidity, it does not capture the full contribution of FP interventions. Namely, the DALY does not include the protection from unplanned pregnancies and birth spacing provided by FP methods. For this reason, PSI continues to measure FP program impact with CYPs, the standard global metric for family planning. CYPs serve as a complementary measure to DALYs averted, each capturing different impacts of PSI's FP products and services.

With the switch to DALYs averted as its key performance metric, PSI made the strategic decision to double its global health impact in five years, from 2007-2011. This goal and the adoption of the DALYs averted metric aligned and motivated PSI's staff operating in 67 countries, either through its own country offices or through locally governed affiliates. The DALYs averted measure was factored into individual performance goals, annual appraisals, and incentive compensation, in addition to the country and regional operating plans.

## Methods

To use DALYs averted as its primary health impact measure, PSI developed several models. These models estimate the impact of a range of preventive and therapeutic interventions for seven specific health areas: HIV/AIDS and other STIs, FP, maternal health (e.g., abortion, clean delivery practice, and micronutrient deficiencies), child health (e.g., nutrition, acute respiratory infection, and diarrhea), malaria, tuberculosis (TB), and cervical cancer. Most model parameters come from one of four data sources: Demographic Health Surveys, United Nations Population Division, WHO, or Multiple Indicator Cluster Surveys. When parameters are unknown, modelers use assumptions based on the published literature or, when necessary, PSI country experiences. Specific details about the model parameters and methodology, together with specific examples of two of PSI's DALYs averted models, are found in another paper in this supplement [43].

Through stochastic modeling, DALYs averted models produce coefficients that are, effectively, the estimated number of DALYs averted by a single product unit (e.g., a condom) in a single country. Each month, PSI estimates health impact from the sales, distribution, and service utilization (collectively referred to forthwith as "distribution") figures reported by each of the country offices. These figures are multiplied by country-level coefficients to determine the number of DALYs averted by each intervention. When products provide multiple years of effectiveness (e.g., long-lasting, insecticide-treated bednets (LLINs), the multi-year impact is credited during the year in which the products are distributed.

PSI also estimates the impact of its product and service promotion efforts done in collaboration with partners. To avoid "over-claiming" impact, PSI counts only partial impact from results stemming from these partnerships, according to policies developed by an internal working group of researchers, modelers, technicians, and implementers. For example, the service referral policy applies to interventions in which PSI generates demand for health services, but does not actually deliver the service, such as adult male circumcision, intrauterine device (IUD) insertions, or HIV testing and counseling. Successful referrals are counted as 50% of the health impact from that service. The significant involvement policy describes how much health impact can be counted from activities in which PSI plays a role in product procurement, communication, and distribution, but is not entirely responsible for product delivery. When PSI participates in a subset of these activities with partners, that subset can be counted toward DALYs averted. PSI has applied this policy to its large-scale, LLIN distribution campaigns in which PSI collaborated with partners to distribute LLINs, but did not actually place the nets directly into the hands of recipients.

Finally, PSI estimates the impact of its behavior change communication (BCC) activities on non-product behaviors and the use of non-PSI brands. Doing so is important for interventions that do not require a product, like the reduction of sexual partners for HIV prevention, or when PSI interventions promote the use of a category of products, such as condoms, rather than simply the use of its own brands. To correlate behavior change with exposure to PSI interventions, PSI uses population-based survey data. When a significant correlation is detected, PSI estimates the size of the population covered, multiplies it by the exposure rate, and estimates the number of new infections averted. These new infections averted are then converted into DALYs averted. Currently, PSI has seven models to estimate DALYs averted through exposure to BCC activities.

Thus, in the most basic terms:

PSI's overall estimate of health impact = (DALYs averted coefficients * PSI distribution) + Service Referral DALYs averted + Significant Involvement DALYs averted + DALYs averted through BCC

Keeping the DALYs averted models current is an ongoing process. As new or more relevant evidence becomes available, PSI revises its models to ensure that its calculations are as accurate as possible. Revised coefficients are applied yearly and adjustments to the models directly affect PSI's bottom line. When appropriate, changes are applied retrospectively; for example, when WHO revises its BOD figures. The health impact in any given year may increase or decrease depending on the models' magnitude of change.

PSI applies the most recent model coefficients when calculating its health impact on a monthly basis. Senior managers at all levels of the organization - global, regional, and country - use these results to monitor progress against intended targets and to guide strategic decision making. They identify areas for improvement and redirect program and funding priorities as needed.

For this paper, PSI's health impact data over the last 10 years (2001-2011) were compiled and analyzed by program area and geographic region in order to demonstrate PSI's progress in achieving its strategic goal of doubling health impact between 2007 and 2011. Showing 10 years of data enables comparison with the years preceding this decision. To generate DALYs averted and standardize annual comparisons, distribution figures for each year were multiplied by the most up-to-date country-level DALYs averted coefficients (2011 coefficients at the time of writing). Following these results, a case study is presented to illustrate how the adoption of DALYs averted as PSI's primary health impact metric affected decision making at the regional level and then within one country in that region.

## Results

Between 2007 and 2011, PSI distributed billions of products and services throughout its worldwide operations. Table 1 presents distribution figures for products and services that accounted for the majority (90%) of the organization's health impact during this time. Two products - LLINs and male condoms - account for 75% of this health impact. Over the course of this five-year period, PSI distributed nearly 114 million LLINs and five billion condoms.

**Table 1:** Product distribution, 2007-2011, in number of units and percentage of DALYs averted

PSI Product or Service	2007 Distribution	2008 Distribution	2009 Distribution	2010 Distribution	2011 Distribution	Total Distribution	% of 2007-2011 DALYs Averted
LLINs	8,539,211	16,367,864	19,025,760	26,564,490	43,413,442	**113,910,767**	46%

Condoms	973,626,161	1,092,370,452	1,175,647,927	1,266,880,272	1,297,766,426	**5,806,291,238**	29%

Malaria pre-packaged therapy (ACTs)	339,593	3,713,502	9,379,305	10,796,682	11,438,863	**35,667,945**	5%

Insecticide retreatment for bednets*	2,770,513	5,636,933	5,022,990	1,428,482	272,854	**15,131,772**	4%

Oral contraceptives	25,060,192	28,595,110	30,187,839	38,336,016	38,272,868	**160,452,025**	2%

HIV counseling and testing	807,813	1,158,011	1,569,736	1,833,415	1,414,591	**6,783,566**	2%

IUDs**	284,127	349,704	618,082	613,595	567,199	**2,432,707**	1%

Basic Care Package for people living with HIV/AIDS	75,648	60,493	352,708	757,187	17,349,693	**18,595,729**	1%

* Either bundled with malaria nets (not LLINs) or distributed alone

** Figures are for IUD distribution only. PSI counts IUD insertions through its service delivery sites differently, applying a higher DALYs averted coefficient.

**Figure 1:**
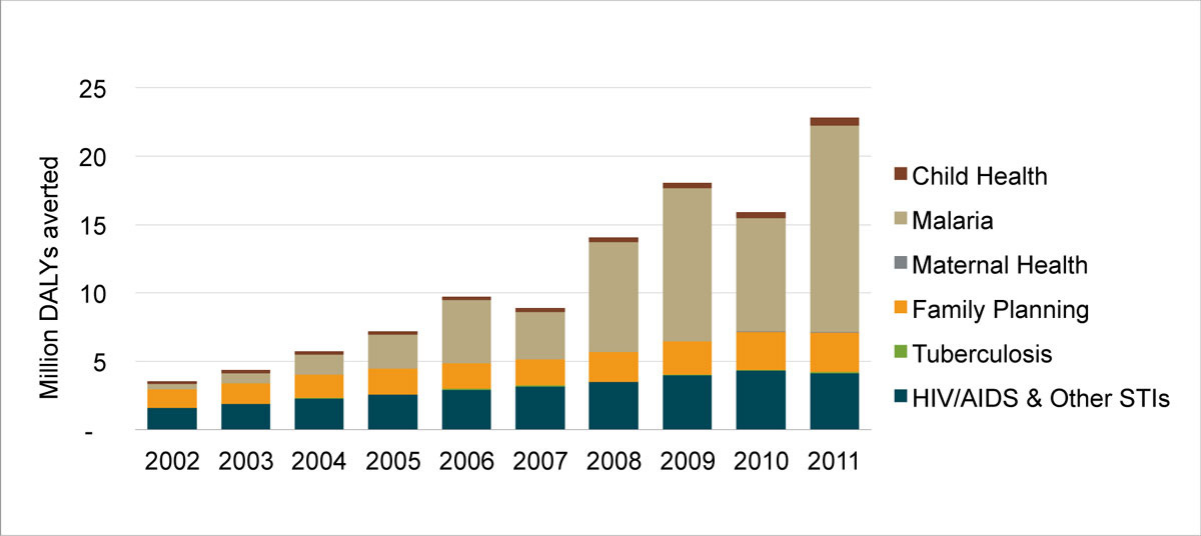
**PSI's global health impact, 2002-2011, in DALYs averted, by health area**.

Figure 1 portrays PSI's health impact as measured in DALYs averted over the course of 10 years. Different colors in the bar charts indicate the health areas in which impact was achieved.

Between 2002 and 2006, PSI averted a total of 30.5 million DALYs, with a compound growth rate of 28.7%. The organization then established the goal of doubling its health impact between 2007 and 2011. During this time there was a marked increase in the total number of DALYs averted. By the end of 2011, PSI had doubled its health impact, averting 22.8 million DALYs in that year. From 2007-2011, 79.7 million total DALYs were averted, with a compound growth rate of 26.6% each year.

Most DALYs averted were in malaria, which accounted for 58% of PSI's overall health impact between 2007-2011. In addition to the high volumes of product distribution, the multi-year effectiveness for LLINs (three years for every net distributed) also helped rapidly increase the number of DALYs averted in malaria, particularly given that this multi-year impact was credited in the year the nets were distributed.

Changes in resource flows influenced PSI's decision making. The Global Fund, for example, invested heavily in LLINs for malaria prevention during this period. Combined with PSI's shift to a BOD-focused strategy, this change in donor priorities made it easier for PSI to recognize that rapid gains in health impact could be achieved by changing its approach from one of sales through commercial channels to one that included free distribution campaigns. A narrower view about the role of PSI as a social marketing organization would have left too much health impact unrealized. As a result, approximately 58% of PSI's health impact in 2007-2011 was from the distribution of free products, namely LLINs and condoms, which means that product sales stopped growing during this period and free distribution became more important for PSI to achieve its bottom line.

The dip in PSI's health impact in 2010 is primarily explained by large LLIN distribution campaigns in countries with lower malaria burdens than those in preceding and subsequent years. Countries with the greatest LLIN distribution over the course of five years were Kenya and the Democratic Republic of Congo with more than 15 million LLINs distributed in each, followed by Madagascar (9.8 million) and Côte d'Ivoire, Uganda, and Cameroon, which each received more than eight million LLINs from PSI distribution channels. By 2011, PSI was also distributing artemisinin-based combination therapies (ACTs) in 12 countries and rapid diagnostic tests in five countries, contributing to additional health impact in malaria. All of these prevention and treatment interventions produced a compound annual growth rate of 44.4% for DALYs averted in malaria between 2007 and 2011.

While a distant second to malaria, HIV/AIDS also experienced steady gains in health impact, increasing at an annual compound growth rate of 7.3% between 2007 and 2011. Increases in HIV DALYs averted were primarily due to extensive condom distribution programs in all three of PSI's African regions. Male circumcision programs also contributed to gains in HIV/AIDS health impact, following its launch in Zambia in 2007 and expansion to another four countries by 2011. The marginal decline in HIV health impact in 2011 is largely attributed to stock outs of free condoms in South Africa.

Health impact within PSI's FP programs steadily increased over the course of 2007-2011, with the greatest gains in the last four years. Condom distribution accounted for the bulk of these increases, which factors into both HIV/AIDS and FP DALYs averted due to condoms' role in dual protection. Grants from an anonymous donor to increase IUD insertions and implants also played a role. The DALYs averted compound growth rate from 2007-2011 in FP was 11.6% each year. During the same period, the organization generated 80.4 million CYPs, with the greatest increase in 2010 and 2011. (Note: CYPs are not featured in Figure 1.)

While PSI achieved small strides with other interventions, such as safe water solution (e.g., chlorination), oral rehydration salts (ORS), and pneumonia pre-packaged therapy (PPT), these products contributed very little to PSI's overall health impact due to limited distribution. They accounted for only 3% of PSI's total DALYs averted between 2007 and 2011. Of these products, PPT shows the greatest promise for health impact, but, so far, PSI's distribution has been limited to just five countries. Additionally, directly observed therapy, short-course (DOTS) for TB shows promise as a contributor to DALYs averted, but PSI's role in distribution is currently limited to only three countries. In terms of nutrition interventions, only PSI/Pakistan has received funding for one nutrition product (*Sprinkles*) at present.

### Strategic changes in Asia and Eastern European region from the use of DALYs averted

PSI's decision to measure organization-wide impact using DALYs averted challenged managers to frame their decisions about strategy, implementation, fundraising, and performance goals in relation to BOD across the regions in which it operates. The Asia and Eastern European (A/EE) region, and one of the countries within it, Laos, offer an instructive example of how the use of DALYs averted affected strategic decisions.

PSI is organized into five regions: 1) West and Central Africa, 2) East Africa, 3) Southern Africa, 4) Asia and Eastern Europe, and 5) Latin America and the Caribbean. Each region develops a strategy that is consistent with PSI's global strategy while respecting varying contexts. Regions are accountable for health impact goals set on an annual basis and monitored throughout the year. As a result, regional directors and country managers develop their intervention strategies and evaluate new funding opportunities based on their potential to contribute to the bottom line in terms of health impact.

**Figure 2:**
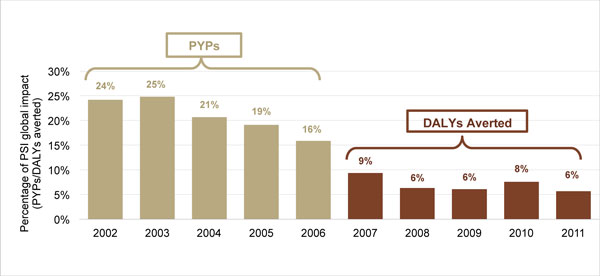
Contribution of PSI's A/EE Region to global impact, 2002-2011, in PYPs and DALYs averted*. *PYPs were used as the performance metric from 2002-06 and DALYs averted from 2007-11.

Under the measurement system of PSI's early years - one based on sales and CYPs - PSI's A/EE region was a relatively high performer, mainly due to large contraceptive social marketing programs in India and Pakistan that sell high volumes of condoms and oral contraceptives. Well-funded programs in Cambodia and Myanmar also made substantial contributions to the regional bottom line. The switch to PYPs had a modest effect on A/EE's relative performance in 2002 and 2003, but as PSI started to scale up malaria programming in sub-Saharan Africa, the relative performance of the region declined (Figure 2).

**Figure 3:**
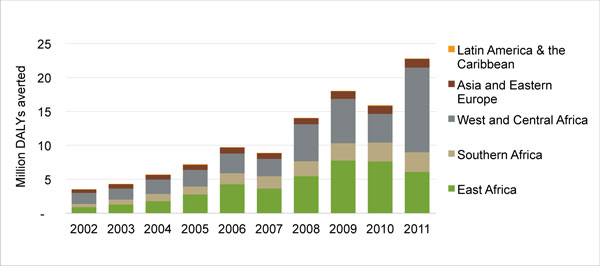
**PSI's global health impact, 2002-2011, in DALYs averted, by region**. *PSI's interventions in Latin America and the Caribbean are smaller than those in other regions of the world, with an average of 65,000 DALYs averted per year between 2002 and 2011. The narrow orange strip at the top of the bar graphs represents this region's contribution to PSI's global health impact.

The switch to DALYs averted had an even greater impact on the region's relative performance. While A/EE averted a total of 5.3 million DALYs from 2007-2011, this achievement represented, on average, just 7% of the organization's overall health impact. The A/EE region's five-year compound growth rate was 11.7%, with the largest increases in child survival and malaria product distribution (30.5% and 20.9%, respectively). By comparison, PSI's country offices in Africa averted more than 74 million DALYs during the same period, with a five-year compound growth rate of 28%. Figure 3 portrays the DALYs averted in both of these regions over the course of 10 years.

Thus, the "success" that Asia and Eastern Europe had experienced from its FP programs when it relied on sales and CYPs was diminished: lower BOD from maternal mortality, perinatal conditions, and HIV/AIDS in Asia meant lower health impact from condoms and modern contraception when PSI adopted DALYs averted as the bottom-line metric. It quickly became clear to managers that meeting the need for HIV prevention and modern contraception in India did not have the same impact as meeting that need in Nigeria where HIV prevalence and maternal mortality are higher.

Some managers in the A/EE region initially viewed the switch to DALYs averted with skepticism, especially those who thought the metric undervalued the region's FP work since it did not capture protection from unplanned pregnancies and the benefits of birth spacing. Others argued that the approach did not adequately value intervening early in an HIV epidemic and with key populations at risk - that is, before the emergence of the high prevalence, generalized epidemic that resulted in high DALYs averted in Africa. Still others, seeing low health impact figures for their programs, paid less attention to PSI's bottom line and continued to focus on process level indicators, which had always been important to the majority of donors and monitored as deliverables under individual interventions.

Managers also realized that many of PSI's programs in A/EE were simply not that relevant to the disease burden in each country. Globally, PSI works heavily in HIV prevention and malaria - despite lower disease burdens in Asia and Eastern Europe - because donor resources are focused on those areas at present. Working in those disease areas in Africa addresses a substantial part of that continent's disease burden. But the shift to DALYs averted pushed the A/EE region toward greater examination of BOD, revealing that country offices needed to be concentrating more on areas like TB diagnosis and treatment as well as non-communicable diseases (NCDs), such as hypertension and smoking-related conditions.

As a result, the A/EE region reformulated its strategy around BOD and included a "relevance" metric in its regional plan in 2010, which meant that a country program's performance would be measured, in part, by the share of the BOD that its interventions could potentially address. The region began identifying funding sources, and designing and implementing interventions in more relevant high BOD areas. The A/EE region also started integrating existing interventions with high BOD interventions; for example, HIV service offerings were combined with TB case detection and treatment. Regional discussions and meetings focused on "relevance" with participation from global experts knowledgeable in higher BOD areas. Expertise in higher BOD areas was also increasingly a factor in the recruitment of new staff at the country program level. Additional gains in strategy have been the introduction of interventions in tobacco control, hypertension, and cervical cancer in A/EE. PSI has also invested resources into expanding its efforts in facility-based interventions, in recognition of the need for quality medical service provision when addressing these new disease areas.

### Impact of the adoption of DALYs averted on PSI/Laos strategy

PSI started operations in Laos in 1999. During its first 10 years, PSI/Laos focused primarily on HIV and malaria prevention, with product distribution of male condoms and LLINs serving as the primary measure of success. This emphasis on sales succeeded in making condoms and malaria nets widely available in the country. Continuing the distribution of existing products was the easiest way to boost sales, rather than investing time in launching products or services in new, unfamiliar health areas.

**Figure 4:**
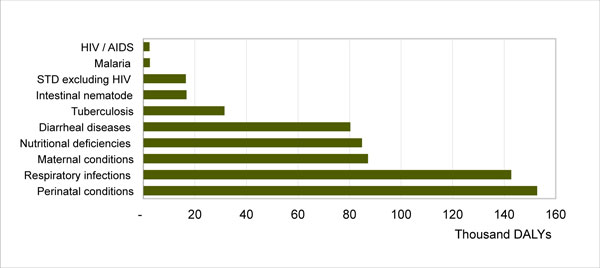
**Burden of disease in Laos (WHO, 2004)**.

The switch to DALYs averted as PSI's primary metric of health impact in 2006 revealed how limited PSI/Laos' impact had been, in terms of reducing the existing BOD. Each year in Laos, HIV/AIDS and malaria cause the loss of 2,398 and 2,571 DALYs, respectively. By contrast, there is a high concentration of DALYs lost every year in four other health areas that PSI/Laos was not working on: lower respiratory infections, especially from pneumonia (134,654 DALYs); unmet need for FP (239,969 DALYs from maternal and perinatal conditions); nutritional deficiencies (84,792 DALYs); and TB (31,448 DALYs)(Figure 4). Confronted with these BOD figures, PSI/Laos began to view its interventions differently and made a fundamental shift in its strategic direction, focusing less on expanding sales and distribution of condoms and LLINs, and more on launching interventions in these four new health areas.

Securing funding to launch such interventions was, and continues to be, a difficult task. The main public health donors for Laos were primarily supporting interventions in certain communicable diseases, such as HIV and malaria, and not providing funds for TB or maternal and child health issues such as pneumonia, nutrition, and diarrheal disease. The PSI/Laos team spent much time and effort trying to interest donors in funding new interventions. By the end of 2010, PSI/Laos secured funding from WHO, Global Fund, and private foundations for three high burden areas: FP, maternal health, and TB. New capacity-building interventions in the private sector aimed to strengthen the Laos health system by providing access to long-term FP methods, misoprostol to reduce postpartum hemorrhage, TB case detection, and DOTS treatment. Monies allocated for these important health areas now comprise approximately half of PSI/Laos' total current funding in country, a major shift from 2010 when all of PSI/Laos' funding was for HIV/AIDS and malaria. Despite this progress, funding for other areas, including pneumonia and diarrheal disease, remain elusive. For those health areas, the PSI/Laos team continues to submit concept notes to donors and is working hard alongside the Laos government, WHO, United Nations, and others to stimulate interest in funding these high BOD areas.

Shifting to more BOD-"relevant" program areas also required a significant adjustment among PSI/Laos' internal staff and external stakeholders who had been accustomed to working primarily on HIV/AIDS and malaria programs. Staff had to develop skills appropriate for the new health area interventions. In some cases, new staff had to be recruited, especially those with clinical skills, to ensure the correct provision of TB DOTS and long-term FP methods.

Paying close attention to DALYs averted rather than product distribution also changed day-to-day program implementation at PSI/Laos. The team reexamined its interventions and how they were implemented, especially questioning the value of ever-expanding sales of existing products for HIV and malaria prevention. The result was a program portfolio of much better targeted and higher quality interventions. For example, HIV/AIDS interventions were increasingly targeted to key populations at higher risk of HIV/AIDS and STIs, such as men who have sex with men and female sex workers. Condoms are still widely distributed, but condom promotion efforts focus more on key populations, those who need them most for prevention of HIV and STIs. Thus, while annual condom distribution numbers dropped considerably for the general population (from about eight million condoms in 2008 to about four million in 2010), rates of condom use among key populations actually rose, according to behavioral surveys [44]. PSI/Laos also improved and diversified its HIV interventions beyond condoms; for example, it launched a network of voluntary counseling and testing sites to increase HIV testing and expand STI screening. On the malaria side, rather than simply trying to expand the sales and distribution of LLINs, PSI/Laos worked closely with the Ministry of Health to re-design the malaria intervention with a focus on more targeted net delivery to villages with the highest rates of malaria.

**Figure 5:**
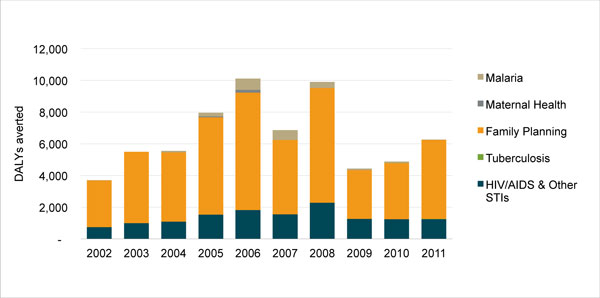
**PSI/Laos' health impact, 2002-2011, in DALYs averted, by health area**.

The health impact generated by PSI/Laos during this transition period reflects the country program's readjustment process. Between 2007 and 2011, PSI/Laos hovered among those PSI countries producing the lowest number of DALYs averted. PSI/Laos recorded a negative overall compound annual growth rate (-2.3%), with annual DALYs averted ranging from 4,439 (the low point, in 2009) to 9,905 (the high point, in 2008). This low impact is largely due to PSI/Laos' more targeted efforts in HIV and more time focused on securing new donor funding in the high BOD areas, actions which resulted in a decreased number of condoms in its "pipeline," and therefore, a reduction in health impact. Since condoms provide dual protection against HIV/AIDS and unplanned pregnancy, this distribution shift meant that PSI/Laos' health impact declined in two health areas, HIV/AIDS and FP. With the shift toward high BOD areas, a focus on long-term contraceptive methods, and additional targeting within HIV/AIDS and malaria, PSI/Laos has slowly, but steadily, increased its DALYs averted. Beginning in 2011, PSI/Laos started to see the impact of its long-term FP method distribution and, while modest, its first DALYs averted from TB interventions. As a result, annual DALYs averted in 2011 increased approximately 50% over its total from 2009, as the focus on high burden areas began to show real impact. Steady growth in overall health impact is expected to continue in coming years (Figure 5).

## Discussion

Using DALYs averted as its bottom-line metric has been valuable for increasing PSI's scope and improving its strategic direction. This measure moved the organization from a counting exercise (sales and PYPs) to a more rigorous and meaningful approach of measuring impact in relation to BOD. Product distribution is still a vital part of the PSI toolbox, but its deployment is now more strategic, based on what the organization learned by applying DALYs averted to measure impact. Using this metric also permitted alignment with the international public health community. PSI can now compare health impact across the different health areas and countries in which it works as well as prioritize its areas of focus across the organization.

PSI's strong focus on malaria prevention and LLIN distribution proved essential in helping the organization achieve its five-year goal of doubling health impact. While this focus was influenced by resource flows, PSI's prioritization of BOD opened up new possibilities for applying its expertise in distribution. These efforts averted more than 36 million DALYs between 2007 and 2011 from the malaria disease burden in the countries where PSI works. Such high impact is due to the scale of the organization's LLIN distribution for malaria prevention, the multiple years of protection provided by LLINs, as well as PSI's work in countries where the burden of malaria is highest, primarily in sub-Saharan Africa.

At the same time, as seen in the case of PSI/Laos, using DALYs averted as the organization's bottom-line metric exposed the need to focus on other high BOD areas and to strategically diversify some country offices' program portfolios - a long-term gain for PSI and for the vulnerable populations experiencing heavy BOD. As a result, all PSI country offices are now more careful when choosing health areas and funding sources.

Setting a clear, performance objective for the organization and tying country office and individual performance goals to the organization's goal of doubling health impact helped align staff around the DALYs averted metric. Country offices where program portfolios were not aligned with BOD, such as PSI/Laos, chose to redirect their efforts to new health areas with higher BOD rather than only concentrating on longstanding interventions in health areas with low disease burden. Even though PSI/Laos experienced losses to health impact in the short term while it made this transition, greater health impact in higher priority health areas is already being recorded. Such growth will likely continue over the medium to long term. These strategic shifts are now being replicated in other parts of the organization. Most importantly, PSI has identified BOD - and an individual country's specific BOD - as one of the main drivers behind its program efforts going forward.

Aligning a large INGO around the DALYs averted metric was not without its challenges, many of which are ongoing. A key challenge is the model development process itself. Building and maintaining tailored DALYs averted models for each intervention proved demanding, particularly given PSI's evidence-based approach and its broad scope of work with over 60 different products and services. The organization's one full-time modeler and two consultants struggled to keep up, especially when new products, interventions, and populations were introduced. Important lessons were also learned about the limitations of specifically tailored models. PSI's DALYs averted models have been constructed for single interventions at a static point in time and cannot be used to consider combined effects, competing risks, or trade-offs between different intervention options.

Moreover, while DALYs averted is a strong metric for capturing information on morbidity and mortality, it does not capture all of PSI's work. DALYs averted does not include the full contribution of PSI's FP interventions, so the organization continues to use CYPs as a complementary measure to estimate the impact of its FP products and services. Capturing the health impact of non-PSI brands and behavior change communication for non-product behaviors (such as number of sexual partners) requires population-based surveys, and behavior change must be attributed to PSI's interventions in order to claim DALYs averted.

The DALYs averted models, like all models, are only as good as the data used to develop them. Data availability is an important limitation. Where data on effectiveness (the measured use of a product or service and its impact on a population) are available, the models take these into account; where data are not available, PSI relies on estimates of efficacy, and measured impact from controlled field trials. At the time of writing, PSI's models used WHO BOD estimates from 2004; they will be updated when country-specific BOD data are available in 2013. Therefore, PSI's decision making in the last five years could have been based on out-of-date estimates of BOD, which would affect its ability to target impact and the relevance of new programs. Reliance on distribution figures as a proxy for product use is a particular challenge; PSI adjusts for wastage in the supply chain (as a general rule, 15% through outlets and 5% through service providers) and compliance in cases where it is required and feasible (e.g., LLINs and ACTs). In most cases, it is impossible to know the profile of populations who purchased or received commodities. As a result, the added benefit of targeting key populations, such as providing female sex workers with condoms, is not taken into account unless a population-based survey is used to estimate the uptake by key populations. Additional data compromises are the inclusion of neighboring country data or regional averages when no local data are available in places where PSI works (e.g., South Sudan and Somaliland). In the absence of reliable data or literature, PSI develops best estimates based on experience for some model parameters, such as supply chain wastage.

Working in new disease areas will increase the pressure on modelers to either produce more new models or adapt existing burden of disease tools from other agencies. Now, PSI is considering new and more efficient methods for modeling health program impact, such as the Lives Saved Tool and the Spectrum Modeling System that consolidates several different models into one integrated package [45-47]. In the meantime, PSI is collaborating with other organizations like Marie Stopes International to align DALYs averted model parameters and assumptions. It is also collaborating with cross-agency working groups to adopt standard metrics and improve their use for decision making. As the responsibility of creating tailored models is likely beyond the capacity of most INGOs, the development of more standardized modeling methodologies, as well as alignment on standard health impact measurement, will benefit the wider global health community.

PSI is also developing methods to calculate cost per DALYs averted in real time so that cost-effectiveness data can be used alongside DALYs averted and BOD to influence decision making. Harmonizing accounting processes and finding methods to apportion costs has been a challenge. When combined with information about the scale of programs and potential reach, cost-effectiveness data will help optimize investment strategies. Such efforts will be the subject of future publications.

There are two important issues that any organization estimating its health impact must consider. The first is attribution. It is important to acknowledge that impact is a shared responsibility and donors and partners working together all contribute to DALYs averted. Shared ownership of health impact should preclude organization-specific claims to attribution. Likewise, organizations should think carefully about the DALYs averted they count, especially when their role is limited to referrals into a larger health system or product distribution on behalf of other partners. PSI addresses attribution through its internal working group, which creates policies to prevent over-claiming health impact. The second issue is substitution, which DALYs averted does not account for; the DALYs averted models treat new users and those who switch product brands or health providers in the same manner. As a result, it is important to track expanding markets for products and services in a different way. PSI strives to expand health markets and increase use through a total market approach, which relies on a different set of performance metrics.

In terms of advocacy, PSI faces a number of challenges due to its new prioritization of BOD. Chief among these is convincing global health funding organizations to support interventions in disease areas outside of those they currently fund. While some funding exists for undernutrition, diarrheal disease, maternal health, and other recognized developing world health conditions, fewer global health donors currently support interventions in tobacco control, cervical cancer, hypertension, and other non-communicable diseases. Regions such as A/EE and Latin America will need to add NCDs to their program portfolios in order to have increased health impact. West and Central Africa may be successful by introducing new child survival programs, including interventions that prevent or treat acute respiratory infections.

PSI has begun educating stakeholders about these concerns, particularly in relation to the BOD in some of the regions it serves, as well as by identifying new donors. While PSI has been successful in getting new donors on board, its strategic shift has also forced its country offices to make hard choices in order to match its work to the actual BOD. In 2010 and 2011, for example, PSI/Laos passed over opportunities for new funding in HIV/AIDS and malaria, preferring to focus on securing donor support in the high burden disease areas. Now, PSI/Laos no longer operates a malaria prevention program. This country office is in the process of scaling up new interventions in FP, reproductive health, and TB, positioning it favorably to achieve greater health impact in the future.

## Conclusion

PSI's adoption of DALYs averted as its measure of health impact had a profound effect on the organization's strategic direction and scope, shifting the focus away from product sales and more toward BOD. Switching to a more robust health metric has produced a more targeted strategy. It also prompted greater diversification in interventions and country programs, and allowed the organization to be responsive to potential funding sources and alternative distribution strategies. Using a bottom-line metric such as DALYs averted can improve interventions and their impact, innovations that global health organizations will need to embrace in response to shifts in the global burden of disease.

## List of abbreviations

PSI: Population Services International; DALY: disability-adjusted life year; BOD: burden of disease; A/EE: Asia and Eastern European region; HIV/AIDS: human immunodeficiency virus/acute immune deficiency syndrome; FP: family planning; STIs: sexually transmitted infections; CYPs: couple-years of protection; SMPH: summary measures of population health; WHO: World Health Organization; INGO: international non-government organization; PYP: Person-Year of Protection; TB: tuberculosis; LLIN: long-lasting, insecticide-treated net; IUD: intrauterine device; BCC: behavior change communication; ACT: artemisinin-based combination therapy; ORS: oral rehydration solution; PPT: pneumonia pre-packaged therapy; DOTS: directly observed treatment, short-course; NCDs: non-communicable diseases

## Competing interests

The authors declare that they have no competing interests.

## Authors' contributions

KL designed the study and drafted the manuscript. BS drafted the regional case study. RG drafted the Laos case study. LN conducted data analysis, prepared all tables and figures, and drafted portions of the Results section. NV conducted the literature review and co-drafted the Background. All authors reviewed and approved the final manuscript.

## Declarations

This article is part of the supplement of *BMC Public Health *Volume 13, Supplement 2, 2013: Use of health impact metrics for programmatic decision making in global health. Population Services International, a registered non-profit organization, provided the funding for the publication of this supplement. The full contents of the supplement are available online at http://www.biomedcentral.com/bmcpublichealth/supplements/13/S2.
